# Dislocation “Bubble-Like-Effect” and the Ambient Temperature Super-plastic Elongation of Body-centred Cubic Single Crystalline Molybdenum

**DOI:** 10.1038/srep22937

**Published:** 2016-03-09

**Authors:** Yan Lu, Sisi Xiang, Lirong Xiao, Lihua Wang, Qingsong Deng, Ze Zhang, Xiaodong Han

**Affiliations:** 1 Beijing Key Lab and Institute of Microstructure and Properties of Advanced Materials, Beijing University of Technology, Beijing, 100124, China; 2 Department of Materials Science, State Key Lab of Si Materials, Zhejiang University, Hangzhou, Zhejiang, 310058, China

## Abstract

With our recently developed deformation device, the *in situ* tensile tests of single crystal molybdenum nanowires with various size and aspect ratio were conducted inside a transmission electron microscope (TEM). We report an unusual ambient temperature (close to room temperature) super-plastic elongation above 127% on single crystal body-centred cubic (bcc) molybdenum nanowires with an optimized aspect ratio and size. A novel dislocation “bubble-like-effect” was uncovered for leading to the homogeneous, large and super-plastic elongation strain in the bcc Mo nanowires. The dislocation bubble-like-effect refers to the process of dislocation nucleation and annihilation, which likes the nucleation and annihilation process of the water bubbles. A significant plastic deformation dependence on the sample’s aspect ratio and size was revealed. The atomic scale TEM observations also demonstrated that a single crystal to poly-crystal transition and a bcc to face-centred cubic phase transformation took place, which assisted the plastic deformation of Mo in small scale.

Nano-sized metals show “unusual deformation” phenomena^1^,^2^,^3^,^4^,^5^,^6^,^7^,^8^,^9^,^10^,^11^ compared to their conventional bulk counterparts, which are derived from their different dislocation nucleation and propagation activities^12^,^13^,^14^,^15^. For face-centred cubic (fcc) metals, full-partial dislocation transitions^16^,^17^,^18^,^19^, dislocation starvation/exhaustion^4^,^20^,^21^,^22^,^23^, liquid-like pseudo-elasticity^3^, source truncation^20^,^21^,^24^,^25^ and the weakest link theory^22^,^25^ have been proposed based on previous experiments.

For body-centred cubic (bcc) metals, the plastic deformation behaviours and the dislocation activities in micro- and nano-sized bcc metals remains unclear, despite many experimental^26^,^27^,^28^,^29^,^30^,^31^,^32^,^33^,^34^ and theoretical^5^,^7^ studies that have been available. In bulk or micro-sized bcc metals, it is well known that screw dislocations dominate the plasticity. The low mobility and self-propagation^7^ of screw dislocations in single crystalline bcc metals readily lead to their entanglement and thus frequently poor elongation rate. It is normally less than 10% in those nanopillars with diameter larger than 300 nm^27^. In recent years, screw dislocation activities was also proposed to be a prevalent plastic behaviour in submicro- and nano-sized bcc metals, in which the intrinsic characters of screw dislocations was supposed to lead to strain hardening^29^,^30^,^32^,^33^ and small power-law slopes^26^,^27^,^28^ as well as poor elongation capabilities. An unusual twinning-assisted plastic deformation behaviour has been reported in a nano-scale bcc tungsten bicrystal^34^ and grain boundaries (GBs) may play a role in the event. However, for nano-scale single crystal bcc metals, plastic deformation behaviours are still largely unknown.

In this work, for the first time, we demonstrate an ambient temperature super-plastic elongation strain above 127% of bcc Mo single crystal nanowire at ambient temperature through *in situ* tensile experiments inside a transmission electron microscope (TEM). The experiments provide direct evidence of the homogeneous elongation capability of a nano-sized bcc metal. A novel plasticity mechanism of dislocation bubble-like-effect was uncovered to be responsible for the ultra-large tensile and super-plasticity occurred in Mo single crystalline nanowires. The dislocation bubble-like-effect derived from the homogeneous dislocation nucleation and escaping processes in small sized bcc metal samples. The effect of the sample’s aspect ratio and size on the homogeneous elongation capability is demonstrated.

## Results

### TEM characterizations of the samples

**Figure 1:**
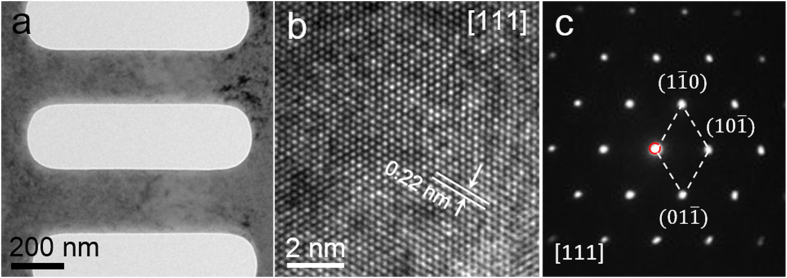
The TEM characterizations of Mo nanowires. (**a**) Bright-field TEM image of the nanowires processed by FIB. (**b**) An HRTEM image of single crystal Mo taken along the [111] direction and (**c**) the corresponding SAEDP.

A focused ion beam (FIB) was used to shape the sample into its final dimensions with a diameter of 100–300 nm for tensile experiments, as shown in Fig. 1a. As seen in Fig. 1b, a high-resolution TEM (HRTEM) image and its corresponding select area electron diffraction pattern (SAEDP) were taken along the [111] direction and reveal that the fabricated nanowires are high quality and contain a low dislocation density.

### Aspect ratio and size effect on the plastic strain of Mo nanowires

**Figure 2:**
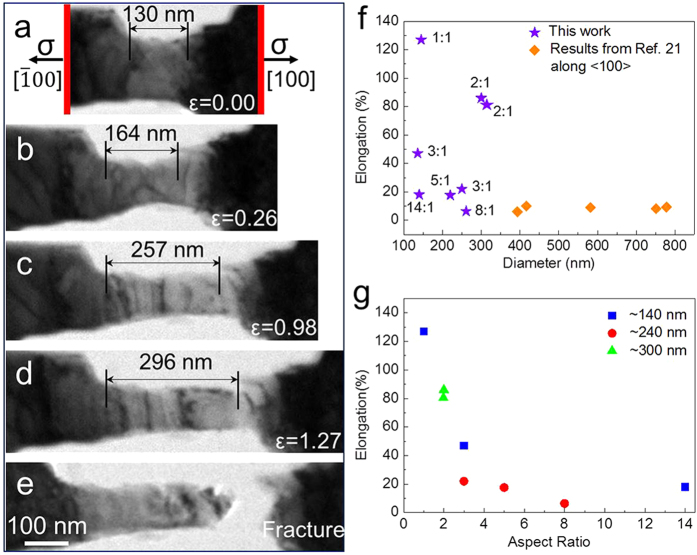
The super-plastic deformation of the Mo nanowires can be approached by optimum design. (**a–e**) A continuous series of bright-field TEM images show the *in situ* tensile process of a single crystal Mo nanowire that is 135 nm in diameter with an aspect ratio of ~1:1. The tensile strain is nearly along the [100] direction. (**f**) The diameter vs. elongation plot. Purple stars are from our experiments, while orange diamonds are from ref. 27. (**g**) The sample’s aspect ratio (ranging from ~1:1 to ~14:1) effects on the elongation capabilities of the nanowires. Blue square, red dot and green triangle indicate a nanowire diameter of ~140 nm, ~240 nm and ~300 nm, respectively.

Figure 2a–e shows a typical example of the homogeneous elongation capability of Mo nanowires using a nanowire with an initial length of 135 nm and a diameter of 130 nm (aspect ratio is approximately 1:1). As the arrows indicate, the tensile direction is nearly along the [100] orientation (with a ~5.4° deviation). Comparing the elongated length *L*_*f*_ (Fig. 2d) with the initial length *L*_*0*_ (Fig. 2a), the nanowire exhibits an unusual homogeneous super-plastic strain of more than 127% at a temperature close to room temperature, which has never been realized before.

A series of Mo nanowires with various diameters were further tested, and the diameter versus elongation relationships is shown in Fig. 2f. For comparison, some reference data are also illustrated in the same figure (the purple stars are from current experiments, and the orange diamonds are from ref. 27). Interestingly, for the nanowires with a diameter greater than 300 nm (orange diamonds), their plastic elongation is as poor as their bulk counterparts; while nanowires with a diameter less than 300 nm (purple stars) exhibit a plastic elongation with an obvious size dependence: the smaller the nanowire diameter, the larger its elongation rate. This indicates that the plastic deformation of the Mo nanowires can be significantly influenced by the nanowire size. However, the scattered distribution of purple stars implies that another intrinsic factor is significantly influencing the plastic strain in the Mo nanowires.

We further investigated the influence of aspect ratio on the plastic strain of the nanowires by using nanowires with various aspect ratios, ranging from ~1:1 to ~14:1. As shown in Fig. 2g, the plastic strain of the nanowires with different aspect ratios are plotted; the blue square, red dot and green triangle represent nanowires with diameters of ~140 nm, ~240 nm and ~300 nm, respectively. For nanowires with diameters of ~140 nm and ~240 nm, the homogenous plastic strain increases as the nanowires’ aspect ratio is reduced (see the blue squares and red dots in Fig. 2g). Unlike submicro-sized Mo specimens that only exhibit ~10% tensile strain^27^, most of the nano-sized Mo display significantly larger elongation rates. By decreasing the nanowires’ aspect ratio to ~2:1 and ~1:1, an ultra-large plastic strain of 86% (see Supplementary Fig. S1) and a super-plasticity of 127% were approached. This demonstrates the substantial impact of aspect ratio on the elongation ability of bcc Mo nanowires. With an optimum aspect ratio, a very unusual homogeneous and ultra-large elongation, even super-plastic rate, can be achieved. By comparing the two nanowires both with aspect ratio of ~3:1, the smaller one exhibits larger strain. The *in situ* TEM images of these two nanowires are shown in Supplementary Fig. S2 and Fig. S3.

### *In situ* observations of dislocation bubble-like-effect

**Figure 3:**
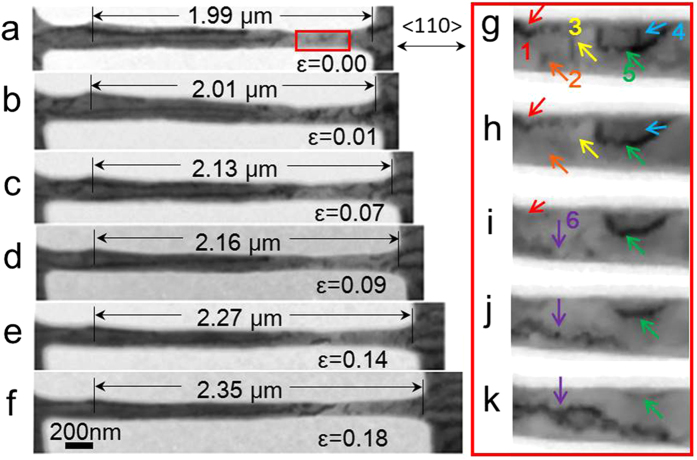
Dislocation bubble-effect during tension. (**a–f**) Bright-field TEM images show the *in situ* tensile process of a single crystal Mo nanowire with a diameter of 140 nm and an aspect ratio of ~14:1. (**g–k**) The evolution process of the dislocations that correspond to the red frame in (**a**); the images are extracted from the video recorded during the tension test. The dislocations are marked with six different colours. The Mo nanowire keeps continuous dislocation nucleation and annihilation processes.

The large and homogeneous plastic elongation of the nano-sized Mo can be explained by such dislocations motion. Figure 3a–f provides a series of TEM images that show the tensile process of a nanowire with 2 μm in length and 140 nm in diameter (an aspect ratio of approximately 14:1). Comparing the elongated length *L*_*f*_ with the initial length *L*_*0*_ (Fig. 3f vs. Fig. 3a), the nanowire exhibits a homogeneous strain of up to 18% before necking occurs. By carefully checking the dislocation behaviours during tensile loading, the nucleation and rapid progression of a dislocation with no obvious entanglement was captured. Figure 3g–k shows the magnified images corresponding to the red framed region in Fig. 3a. These images were extracted from the video recorded during the tensile test, and the black dot/line contrasts, i.e., dislocations, are marked by different coloured arrows and numbers. During the tensile loading process, dislocations “1” through “4” moved quickly and escaped from the surface of the nanowire, as shown in Fig. 3g,h. With further tensile elongation, a new dislocation “6” nucleated, as shown in Fig. 3i. In the same manner, dislocation “6” also moved as the strain increased, whereas dislocation “5” escaped (Fig. 3j) from the surface of the nanowire. This *in situ* tensile experiment on a Mo nanowire revealed continuous dislocation nucleation and annihilation processes, i.e., a novel dislocation bubble-like-effect mechanism. During the tensile process, the dislocation nucleation rate was roughly equivalent to the annihilation rate. Sometimes the dislocation nucleation rate might be higher or lower than the annihilation rate but it only lasted for a short while. Once the balance was broken, i.e., the dislocation nucleation rate was higher or lower than the annihilation rate for a long time, work hardening or dislocation starvation would happen and necking of the tensile samples would follow.

The dislocation bubble-like-effect is somewhat similar to the dislocation starvation mechanism in fcc metals, which suggests fast dislocation annihilation rates in small-sized fcc metals. The difference is that the dislocation bubble-like-effect mechanism needs continuous and homogeneous dislocation nucleation activities. In contrast, dislocation starvation normally exhausts the dislocation source and cannot approach super-plasticity in fcc metals. The homogeneous dislocation nucleation here means that the dislocation nucleation and propagation activities are spatially homogeneous to ensure a homogeneous elongation and even super-plasticity. The spatially inhomogeneous dislocation nucleation and propagation would lead to local shear, dislocation entanglement or starvation; consequently early necking could happen and impede super-plastic deformation behaviours. The dislocation bubble-like-effect phenomenon is frequently observed in our experiments and often leads to an extensive plastic elongation of bcc Mo nanowires.

### Single crystal to poly-crystal transformation

**Figure 4:**
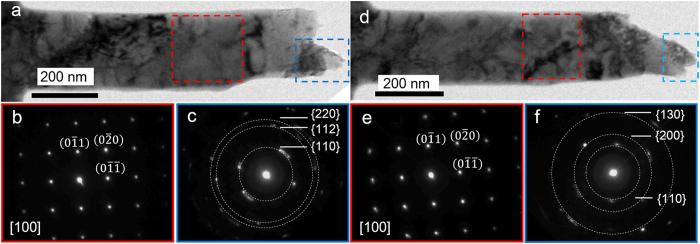
Single crystal to poly-crystalline transformation. (**a,d**) Typical TEM images showing two fractured nanowires (**b,c,e,f**) and SAEDPs corresponding to the red and blue framed regions in (**a,d**). The white dotted circles in (**c,f**) mark the splitting diffraction spots, and the measured angles between the splitting diffraction spots are below 20°.

**Figure 5:**
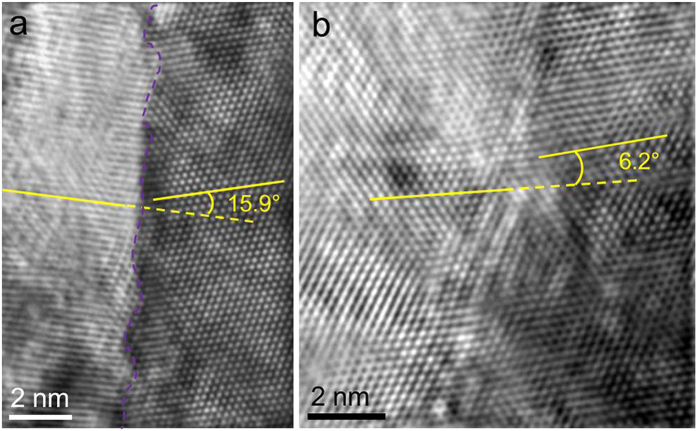
HRTEM observation of the GB formation induced by tensile deformation. The images are taken along the [111] direction. (**a**) A GB with angle of 15.9° was observed near the fracture region. The GB was drawn with purple dash line. (**b**) A GB with angle of 6.2° was observed during necking.

In addition to the dynamic dislocation processes, a single crystal to poly-crystal transformation event was also observed. Figure 4a,d show two typical TEM images that were captured after a nanowire fracture. The nanowire was stretched along [011] direction. The bright-dark contrast near the fracture surface indicates that the nanowire underwent a severe plastic deformation. Figure 4b,e present the SAEDPs taken from the red frame regions in Fig. 4a,d, respectively. The SAEDPs show typical single crystalline features. However, the SAEDPs that were taken close to the fracture regions (blue frames in Fig. 4a,d) of the nanowires possess splitting diffraction spots (Fig. 4c,f) that clearly indicate poly-crystalline features with severe deformation. The white dotted circles are drawn to clarify the splitting spots on the same diffraction ring. HRTEM observations further confirm the poly-crystallization transformation in tensile-fractured nanowires. Figure 5a,b are two typical HRTEM images taken along the [111] axis and were captured during the necking process and post-fracture, respectively. In Fig. 5a, a GB with a mis-orientation angle of 15.9° was revealed, while in Fig. 5b, a GB angle was approximately 6.2°. The small-angle GB formation in the nanowires indicates that multiple dislocation sources were activated.

The small size and large free surface enable the dislocations to continuously nucleate. The nucleated dislocations subsequently glide to the surface and annihilated prior to entanglement. Through this process, small angle grain boundaries could form with possible dislocation interactions derived from dislocations on different slip systems. The interaction of dislocations could form locks or pin sites to pile up dislocations and form small angle GBs^12^,^35^,^36^,^37^.

### Bcc to fcc phase transformation

**Figure 6:**
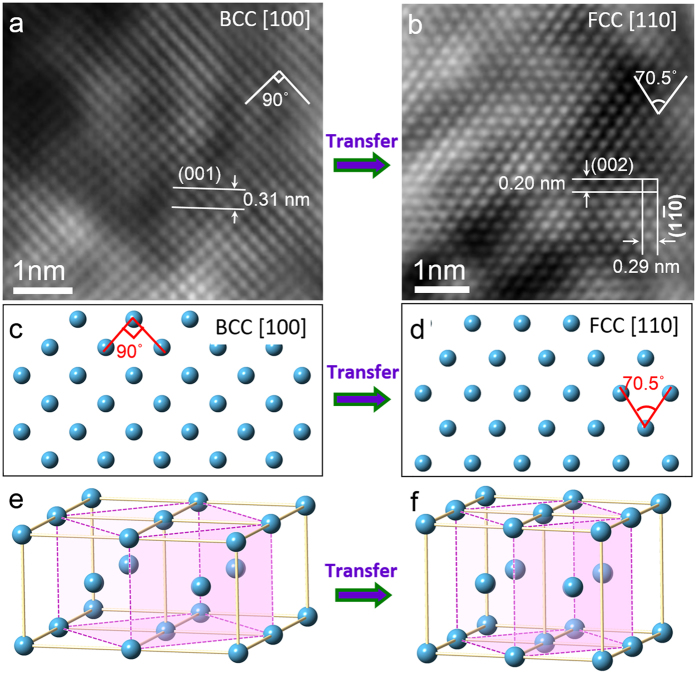
Bcc structure to fcc structure transformation. (**a**) HRTEM image of a Mo nanowire that was captured along the [100] direction; it shows a typical bcc lattice. (**b**) HRTEM image of Mo nanowire that was taken along the [100] direction; it shows typical fcc lattice features. (**c**) Atomic model of bcc Mo that project along the [100] direction. (**d**) Atomic model of the fcc Mo that project along the [110] direction. (**e,f**) 3D atomic structure show the of the transformation path from bcc Mo to fcc Mo.

Accompanying the poly-crystallization process, the phase transformation from bcc to fcc also occurred. Figure 6a shows a typical HRTEM image taken along the [100] axis in the region of a fracture. The lattice exhibits a square shape, which is consistent with bcc Mo. However, in addition to the square-shaped lattice, parallelogram-shaped lattices were also observed in the same view, as revealed in Fig. 6b. The measured inter-planar angle is exactly 70.5°, which cannot be interpreted as a bcc structure, and has no low index plane with an inter-planar angle of 70.5°. This is, however, consistent with an fcc structure containing a 70.5° inter-planar angle between (111) and (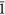
11) planes, as viewed along the [110] axis. This bcc to fcc transformation is confirmed by measuring the inter-planar spacing of the {002} and {110} planes, which are 0.20 nm and 0.29 nm, respectively, and is consistent with fcc Mo (lattice constant is 0.403 nm)^38^. The transformation is analogous to the Bain lattice deformation process through “principle-axis” straining^39^, which has traditionally been used to model a martensitic transformation from an fcc lattice to a bcc lattice^40^. An atomic model of the bcc to fcc transformation is shown in Fig. 6c–f. Figure 6c,d show images projected along the bcc [100] direction and along fcc [110]. A 3D atomic model of four bcc unit cells with a lattice constant ratio *a*:*b*:*c* = 1:1:1 is shown in Fig. 6e. The pink dashed lines and pink shade planes are drawn to indicate a face-centered tetragonal (fct) unit cell. When subjected to a stress, the fct unit cell changes towards fcc cell. Once the original bcc lattice changes into *a*:*b*:*c* = 1:1: 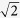, then the bcc lattice to fcc lattice transformation is realized, as show in Fig. 6f. The fcc cell in Fig. 6f is marked by pink dashed lines and pink shade planes. The lattice constant for the new fcc phase is *a’*:*b’*:*c’* = 1:1:1. It should be noted that the (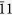
0) and (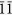
0) planes in the bcc structure transform into the (100) and (010) planes in the fcc structure, respectively.

## Discussion and Conclusions

The ultra-large and super-plastic straining capabilities of Mo nanowires at ambient temperature may have several origins. First, the diameters are 100–300 nm, and the thicknesses of all specimens are approximately 100 nm. The large free surface with step sources helps the continuous nucleation of dislocations, and the small size with large fraction of surfaces favours the dislocation annihilation prior to entanglement, which leads to the super-plastic deformation. The nanowires can thus sustain a stable dislocation density, whereby the balance between dislocation generation and escaping is maintained. Once the balance was broken, necking of the tensile samples will follow. Second, the homogeneous elongation ability must be mediated by whether the continuous dislocation nucleation and movement are homogeneously distributed throughout different sites and multiple slip systems. For nanowires with large aspect ratios, plastic deformation is more likely driven by dislocations that form on the same slip system^41^. These dislocations concentrate in the shear region, leading to nanowire failure via localized shear with a small homogeneous elongation. As for the nanowires with small aspect ratios, more spaces along the circumference could potentially serve as dislocation nucleation sites. The dislocations also have higher chances to be activated on multiple slip systems, leading to a large and homogeneous elongation before failure.

Though the aspect ratio effect on the plastic strain was observed in both of nanoscale single crystalline bcc metal and the macroscale dog-bone tensile specimens of copper and Al-Mg alloys^42^,^43^, the deformation mechanisms are quite distinctive. In the Cu tensile specimens, the homogeneous elongations before necking were rarely influenced by the aspect ratio^42^, while the fracture strain (after necking) was aspect ratio depended. As to the superplastic alloy Al–4 5Mg^43^, the aspect ratio dependence of the plastic strain was due to the accommodation with the matrix^43^. In the current case of room temperature super-plastic single crystalline Mo, the homogeneous elongation behaviours before necking of Mo nanowires are various with different diameters and aspect ratios. The large plastic strain and super-plastic elongation were due to the continuous nucleation/propagation of dislocations and the subsequent dislocation annihilation on the surface for a constant dislocation density. The size, aspect ratio and surface together created a bubble-like-effect of dislocations, and derived the super-plastic deformation ability of single crystal Mo at small scale.

In addition, the corners between the tensile samples and the supporting frames influenced the plastic strain. The stress prefers to concentrate at the corners, which promotes the crack initiation. However, the confinement effect of the sample’s frame-window substrate could help to delay nucleation of cracks and localized shearing.

In summary, using our recently developed *in situ* TEM mechanical devices, tensile experiments were performed on bcc Mo nanowires with various aspect ratios and sizes. We revealed a novel dislocation bubble-like-effect mechanism which may lead to an ultra-large plasticity and super-plastic deformation behaviours of nano-sized bcc metals. The dislocation bubble-like-effect consists of a continuous and homogeneous dislocation nucleation process and a continuous dislocation annihilation event. The dislocation bubble-like-effect process can be optimized and significantly influenced by the bcc metallic nanowires’ aspect ratio and size. By proper design and optimization, a super-plastic elongation rate above 127% of a Mo nanowire was demonstrated. These results provide opportunities for easier processing of bcc metals on the small scale and can be useful in designing nanostructures with bcc metals that possess both high strength and ductility.

## Experimental Procedures

Single crystalline Mo specimens were mechanically polished to a thickness of approximately 50 μm before twin-jet electropolishing. Then, small strip samples were cut from the edge of the twin-jet electropolished sample and were sequentially transferred to a homemade TEM tensile device^44^,^45^,^46^. Subsequently, a focused ion beam (FIB) was used to shape the sample into its final dimensions with a diameter of 100–300 nm for tensile experiments. Finally, carefully fix the whole device with the prepared sample into a Gatan double tilt heating holder. The *in situ* and real-time tensile tests and microstructure investigations of the Mo nanowires were conducted in a JEOL-2010 and a JEOL-2010 F TEM at 200 kV. The experimental temperature was below 100 °C (~0.13 T_m_), which is far below the melting point of Mo.

## Additional Information

**How to cite this article**: Lu, Y. *et al.* Dislocation “Bubble-Like-Effect” and the Ambient Temperature Super-plastic Elongation of Body-centred Cubic Single Crystalline Molybdenum. *Sci. Rep.* **6**, 22937; doi: 10.1038/srep22937 (2016).

## Supplementary Material

Supplementary Information

## References

[b1] Uchic M. D., Dimiduk D. M., Florando J. N. & Nix W. D. Sample dimensions influence strength and crystal plasticity. Science 305, 986–989 (2004). 15310897 10.1126/science.1098993

[b2] Csikor F. F., Motz C., Weygand D., Zaiser M. & Zapperi S. Dislocation avalanches, strain bursts, and the problem of plastic forming at the micrometer scale. Science 318, 251–254 (2007). 17932293 10.1126/science.1143719

[b3] Yue Y. H. *et al.* Crystalline liquid and rubber like behavior in Cu nanowires. Nano Lett. 13, 3812–3816 (2013). 23898785 10.1021/nl401829e

[b4] Greer J. R. & Nix W. D. Nanoscale gold pillars strengthened through dislocation starvation. Phy. Rev. B 73, 245410 (2006).

[b5] Li S. Z. *et al.* High-efficiency mechanical energy storage and retrieval using interfaces in nanowires. Nano Lett. 10, 1774–1779 (2010). 20369897 10.1021/nl100263p

[b6] Fujita T. *et al.* Atomic origins of the high catalytic activity of nanoporous gold. Nat. Mater. 11, 775–780 (2012). 22886067 10.1038/nmat3391

[b7] Weinberger C. R. & Cai W. Surface-controlled dislocation multiplication in metal micropillars. Proc. Natl. Acad. Sci. 105, 14304–14307 (2008). 18787126 10.1073/pnas.0806118105 PMC2567194

[b8] Wang L. H., Zhang Z. & Han X. D. *In situ* experimental mechanics of nanomaterials at the atomic scale. NPG Asia Mater. 5, e40 (2013).

[b9] Bao P. *et al.* Atomic-scale observation of parallel development of super elasticity and reversible plasticity in GaAs nanowires. Appl. Phys. Lett. 104, 021904 (2014).

[b10] Du Z. *et al.* Size effects and shape memory properties in ZrO_2_ ceramic micro- and nano-pillars. Scripta Mater. 101, 40–43 (2015).

[b11] Hassani-Gangaraj S. M., Cho K. S., Voigt H.-J. L., Guagliano M. & Schuh C. A. Experimental assessment and simulation of surface nanocrystallization by severe shot peening. Acta Mater. 97, 105–115 (2015).

[b12] Liu P., Mao S. C., Wang L. H., Han X. D. & Zhang Z. Direct dynamic atomic mechanisms of strain-induced grain rotation in nanocrystalline, textured, columnar-structured thin gold films. Scripta Mater. 64, 343–346 (2011).

[b13] Wang L. H. *et al.* Grain rotation mediated by grain boundary dislocation in nanocrystalline platinum. Nat. Comm. 5, 4402 (2014). 10.1038/ncomms5402 PMC4109021 25030380

[b14] Chen Y. *et al.* Determination of Young’s modulus of ultrathin nanomaterials. Nano Lett. 15, 5279–5283 (2015). 26189461 10.1021/acs.nanolett.5b01603

[b15] Zhu T., Li J., Samanta A., Kim H., G. & Suresh S. Interfacial plasticity governs strain rate sensitivity an ductility in nanostructured metals. Proc. Natl. Acad. Sci. 104, 3031–3036 (2007). 17360604 10.1073/pnas.0611097104 PMC1805608

[b16] Chen M. W. *et al.* Deformation twinning in nanocrystalline aluminum. Science 300, 1275–1277 (2003). 12714676 10.1126/science.1083727

[b17] Zhu Y. T., Liao X. Z. & Wu X. L. Deformation twinning in nanocrystalline materials. Prog. Mater. Sci. 57, 1–62 (2012).

[b18] Yue Y. H. *et al.* Quantitative evidence of crossover toward partial dislocation mediated plasticity in copper single crystalline nanowires. Nano Lett. 12, 4045–4049 (2012). 22731885 10.1021/nl3014132

[b19] Wang L. H. *et al.* *In situ* observation of dislocation behavior in nanometer grains. Phys. Rev. Lett. 105, 135501 (2010). 21230786 10.1103/PhysRevLett.105.135501

[b20] Kiener D. & Minor A. M. Source truncation and exhaustion: insights from quantitative *in situ* TEM tensile testing. Nano Lett. 11, 3816–3820 (2011). 21793497 10.1021/nl201890s PMC3172822

[b21] Oh S. H., Legros M., Kiener D. & Dehm G. *In situ* observation of dislocation nucleation and escape in a submicrometre aluminium single crystal. Nat. Mater. 8, 95–100 (2009). 19151703 10.1038/nmat2370

[b22] Norfleet D. M., Dimiduk D. M., Polasik S. J., Uchic M. D. & Mills M. J. Dislocation structures and their relationship to strength in deformed nickel microcrystals. Acta Mater. 56, 2988–3001 (2008).

[b23] Benzerga A. A. An analysis of exhaustion hardening in micron-scale plasticity. Int. J. Plast. 24, 1128–1157 (2008).

[b24] Rao S. I. *et al.* Athermal mechanisms of size-dependent crystal flow gleaned from three-dimensional discrete dislocation simulations. Acta Mater. 56, 3245–3259 (2008).

[b25] Parthasarathy T. A., Rao S. I., Dimiduk D. M., Uchic M. D. & Trinkle D. R. Contribution to size effect of yield strength from the stochastics of dislocation source lengths in finite samples. Scripta Mater. 56, 313–316 (2007).

[b26] Scheneider A. S. *et al.* Correlation between critical temperature and strength of small-scale bcc pillars. *Phys*. Rev. Lett. 103, 105501 (2009). 10.1103/PhysRevLett.103.105501 19792329

[b27] Kim J. Y., Jong D. C. & Greer J. R. Tensile and compressive behavior of tungsten, molybdenum, tantalum and niobium at the nanoscale. Acta Mater. 58, 2355–2363 (2010).

[b28] Schneider A. S., Clark B. G., Frick C. P., Gruber P. A. & Arzt E. Effect of orientation and loading rate on compression behavior of small-scale Mo pillars. Mater. Sci. Eng. A 508, 241–246 (2009).

[b29] Huang L. *et al.* A new regime for mechanical annealing and strong sample-size strengthening in body centred cubic molybdenum. Nat. Comm. 2, 547 (2011). 10.1038/ncomms1557 22109521

[b30] Brinckmann S., Kim J. Y. & Greer J. R. Fundamental differences in mechanical behavior between two types of crystals at the nanoscale. Phys. Rev. Lett. 100, 155502 (2008). 18518121 10.1103/PhysRevLett.100.155502

[b31] Bei H. *et al.* Compressive strengths of molybdenum alloy micro-pillars prepared using a new technique. Scripta Mater. 57, 397–400 (2007).

[b32] Bei H., Shim S., Pharr G. M. & George E. P. Effects of pre-strain on the compressive stress-strain response of Mo-alloy single-crystal micropillars. Acta Mater. 56, 4762–4770 (2008).

[b33] Greer J. R., Weinberger C. R. & Cai W. Comparing the strength of fcc and bcc sub-micrometer pillars: compression experiments and dislocation dynamics simulations. Mater. Sci. Eng. A 493, 21–25 (2008).

[b34] Wang J. *et al.* *In situ* atomic-scale observation of twinning-dominated deformation in nanoscale body-centred cubic tungsten. Nat. Mater. 14, 594–600 (2015). 25751073 10.1038/nmat4228

[b35] Wang L. H. *et al.* *In situ* atomic-scale observation of continuous and reversible lattice deformation beyond the elastic limit. Nat. Comm. 4, 2413 (2013). 10.1038/ncomms3413 PMC3778763 24022231

[b36] Murayama M., Howe J. M., Hidaka H. & Takaki S. Atomic-level observation of disclination dipoles in mechanically milled nanocrystalline Fe. Science 295, 2433–2435 (2002). 11923534 10.1126/science.1067430

[b37] Sun L., Muszka K., Wynne B. P. & Palmiere E. J. The effect of strain path reversal on high-angle boundary formation by grain subdivision in a model austenitic steel. Scripta Mater. 64, 280–283 (2011).

[b38] Häglund J., Guillermet A. F., Grimvall G. & Körling M. Theory of bonding in transition-metal carbides and nitrides. Rhys. Rev. B 48, 11685 (1993). 10.1103/physrevb.48.11685 10007503

[b39] Luo W. D., Roundy D., Cohen M. L. & Morris J. W. Jr. Ideal strength of bcc molybdenum and niobium. Phys. Rev. B 66, 094110 (2002).

[b40] Bain E. C. & Dunkirk N. Y. The nature of martensite. Trans. Am. In. Min. Metall. Eng. 70, 25–47 (1924).

[b41] Wu Z. X., Zhang Y. W., Jhon M. H., Gao H. J. & Srolovitz D. J. Nanowire failure: long = birttle and short = ductile. Nano Lett. 12, 910–914 (2012). 22214242 10.1021/nl203980u

[b42] Zhao Y. H. *et al.* Influence of specimen dimensions on the tensile behavior of ultrafiine-grained Cu. Scripta Mater. 59, 627–630 (2008).

[b43] Bate P. S., Ridley N. & Sotoudeh K. Effect of gauge length in superplastic tensile tests. Mater. Sci. Tech. 24, 1265–1270 (2008).

[b44] Wang L. H., Zhang Z., Ma E. & Han X. D. Transmission electron microscopy observation of dislocation annihilation and storage in nanograins. Appl. Phys. Lett. 98, 051905 (2011).

[b45] Deng Q. S. *et al.* Uniform tensile elongation in framed submicron metallic glass specimen in the limit of suppressed shear banding. Acta Mater. 59, 6511–6518 (2011).

[b46] Yue Y. H., Liu P., Zhang Z., Han X. D. & Ma E. Approaching the theoretical elastic strain limit in copper nanowires. Nano Lett. 11, 3151–3155 (2011). 21766832 10.1021/nl201233u

