# Supplementary material for: Dislocation “Bubble-Like-Effect” and the Ambient Temperature Super-plastic Elongation of Body-centred Cubic Single Crystalline Molybdenum
Source: Sci Rep. 2016 Mar 9;6:22937. doi: 10.1038/srep22937 (PMC4783708; doi:10.1038/srep22937)
Supplement: Supplementary Information [file srep22937-s1.pdf]

Supplementary Information for:

Dislocation "Bubble-Like-Effect" and the Ambient Temperature Super-plastic Elongation of Body-centred Cubic Single Crystalline Molybdenum

Yan Lu[1], Sisi Xiang[1], Lirong Xiao[1], Lihua Wang[1,*], Qqingsong Deng[1], Ze Zhang[1,2], Xiaodong Han[1,*]

[1] Institute of Microstructure and Properties of Advanced Materials, Beijing University of Technology, Beijing, 100124, China

[2] Department of Materials Science, State Key Lab of Si Materials, Zhejiang University, Hangzhou, Zhejiang, 310027, China

Corresponding author: wlh@bjut.edu.cn; xdhan@bjut.edu.cn

Super-plastic elongation of single crystal Mo nanowire. The plastic strain measurement exclude the necking region. The nanowire with 300 nm in diameter and aspect ratio of ~2:1 is demonstrated in Fig. S1. The *in situ* TEM images of single crystalline Mo nanowire with 136 nm and 243 nm in diameter are shown in Fig. S2 and Fig. S3, respectively.

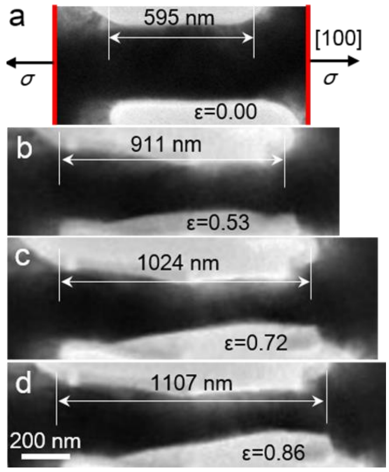

Figure S1. A series of bright-field TEM images of a nanowire with an aspect ratio of ~2:1. The nanowire is with an initial width of ~300 nm and a length of 595 nm. The elongation is ~86% before fracture.

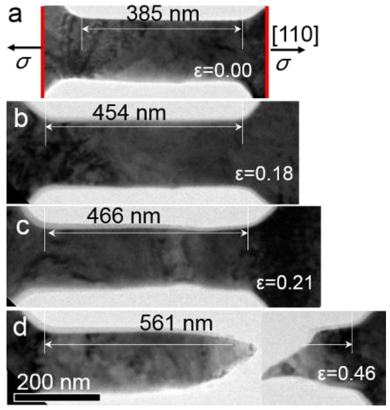

Figure S2. The *in situ* tensile progress of Mo nanowire that is 136 nm in diameter and 385 nm in initial length with aspect ratio of ~3:1. The final length of nanowire is measured to be 561 nm; the plastic elongation is 46%.

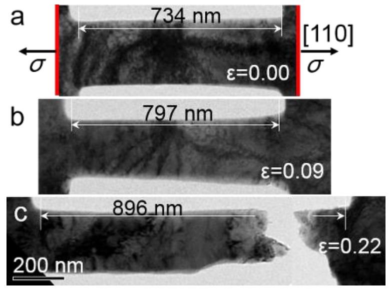

Figure S3. The in situ TEM images of a nanowire with 243 nm in diameter, 734 nm in initial length and aspect ratio of ~3:1. The final length excluding necking region is 896 nm; the plastic elongation is 22%.
